# Work Resumption after a Fixed-Term Disability Pension: Changes over Time during a Period of Decreasing Incidence of Disability Retirement

**DOI:** 10.3390/ijerph18094618

**Published:** 2021-04-27

**Authors:** Mikko Laaksonen

**Affiliations:** Finnish Centre for Pensions (ETK), Eläketurvakeskus, 00065 Helsinki, Finland; mikko.laaksonen@etk.fi; Tel.: +358-29-411-2156

**Keywords:** disability retirement, return to work, work ability, vocational rehabilitation, mental disorders, musculoskeletal diseases

## Abstract

The incidence of disability retirement in Finland has sharply decreased over the last ten years. At the same time, the share of fixed-term pensions has increased to cover more than half of all new disability pensions. This study examined the efficiency of fixed-term disability pensions under these changing circumstances with the aim of addressing the following research questions: are fixed-term disability pensions more often converted to permanent pensions, and how have the changes affected return to work? The study was based on register data of Finnish residents aged 25–62 whose fixed-term disability pension started in 2006 (*n* = 10,177) or 2015 (*n* = 7918). Of the fixed-term disability pensions starting in 2006, 58 percent were converted to a permanent disability pension within the next four years. In 2015, the corresponding figure was 41 percent. Return to work increased from 24 to 30 percent. In addition, ending up in some other state (most often unemployment) increased, and, more often, fixed-term disability pensions continued for more than four years. Transferring to a permanent disability pension decreased more among the younger, those with a lower education, and those suffering from mental disorders. The results were not notably affected by changes in the characteristics of fixed-term disability pension recipients (e.g., demographic and occupational characteristics and rehabilitation) or the selection of applicants into a fixed-term or a permanent pension. Return to work increased more among men, the older age groups, those with a fixed-term disability pension due to somatic diseases, and those who had received vocational rehabilitation. Adjusting for the composition of fixed-term disability pensioners increased the differences between the study years, and controlling for the selection into a fixed-term pension further widened the differences. In conclusion, a decreasing proportion of fixed-term disability pensions are converted to permanent pensions, but this is only partly reflected in increased return to work. Further efforts are needed to support work resumption after a fixed-term disability pension to avoid the situation where people drop out from disability pension but are resting on unemployment and other benefits.

## 1. Introduction

Many western countries have experienced a substantial increase in life expectancy and a decrease in fertility in recent decades. To increase the share of the active working population relative to retirees, many governments have raised the statutory retirement age [1]. However, it is equally important to ensure that people are able to continue to work until that age. As aging workers inevitably suffer from illnesses and chronic conditions, it is important to improve the possibilities to return to work after an illness and to avoid a permanent exit from the workforce [2,3].

In many countries, disability benefits are time-limited, and the need for their continuation is assessed at regular intervals [4]. In Finland, sickness allowance covers short-term work disability lasting up to one year. In the case of an extended period of loss of work ability, a disability pension may be granted [5]. However, if it is considered possible that the employee’s work ability can be restored through treatment or rehabilitation, the pension is granted for a fixed term. Often, the fixed-term disability pension is continued after the initial period. There is no maximum duration for the fixed-term pension, but if there are no good grounds (such as ongoing treatment or rehabilitation) for extending it further, a final decision between a permanent disability pension and a return to the labor market should be normally made within two years. A large proportion of fixed-term disability pensions are converted into permanent pensions at some point [6].

Since the early 2000s, the incidence of disability retirement in Finland has decreased [7]. At the same time, the proportion of fixed-term disability pensions has increased. These trends may reflect improved work ability or increased societal pressure to extend working lives, leading to stricter (conscious or unconscious) evaluation criteria, particularly when a permanent pension is considered. As the purpose for the fixed-term disability pension scheme is to allow return to work after an extended work disability, it is crucial to evaluate whether such goals are reached. Does the increasing share of fixed-term pensions mean that they are converted to permanent disability pensions more often than before, or do they lead to positive employment outcomes?

The use of fixed-term disability pensions varies by age and diagnosis [8]. Fixed-term pensions are highly common in the younger age groups and among those diagnosed with a mental disorder. As disability pensions in the younger age groups are typically based on mental disorders, these two dimensions also overlap. A previous Finnish study [6] found that younger age and higher education increased the probability of return to work after a fixed-term disability pension, but the gaps between men and women, private and public sector employees, and occupational classes were relatively small. Return to work was more probable after a fixed-term disability pension due to musculoskeletal diseases or an injury and less probable when the fixed-term disability pension was based on mental disorders. Return to work was more likely among those who were employed before their fixed-term disability pension and among those who participated in vocational rehabilitation.

Return to work after a fixed-term disability pension has also been examined elsewhere. A German interview study found that a very small proportion of fixed-term disability pensioners returned to work, and it was clearly more common to receive a permanent pension [9]. Return to work seemed to be more likely among the younger and those with a shorter fixed-term pension, but due to the low number of those returning to work, the results are tentative. Another German study, using a 7-year register-based follow-up, found that the probability of return to work was increased by younger age, a somatic rather than a mental illness, participation in medical rehabilitation, and being employed before the disability pension began [10]. A Norwegian study examined the occupational status among rehabilitation allowance recipients (intended for workers who have been sick for more than one year) over three years [11]. Overall, 27 percent of the participants returned to work. Earlier work experience, younger age and having other members in the household were found to predict return to work. In contrast, gender and diagnosis (musculoskeletal diseases versus mental disorders) were not associated with the probability of return to work.

This study examined transferring to a permanent disability pension and returning to work over a period of four years among those who started their fixed-term disability pension in 2006 or 2015. Changes in the characteristics of the fixed-term disability pensioners were considered to control for possible changes in their composition. Additionally, the possible selection effect due to the increasing share of disability pensions granted for a fixed term rather than a permanent basis was controlled for.

## 2. Materials and Methods

### 2.1. Data

A nationwide register of the Finnish Centre for Pensions was used to identify all persons who started receiving a fixed-term disability pension in 2006 or 2015. The register includes all pension recipients from the earnings-related pension scheme, which is the primary pension scheme in Finland. It covers all persons who have accrued pension through employment or self-employment but not those who have never worked and therefore receive only a national pension. The lower age limit for the disability pension is 18 years and the upper limit is 62 years, after which only old-age pensions are granted. However, those who were younger than 25 were excluded, because as a result of the 2005 pension reform, people with a very short working history also became eligible for the earnings-related pension. Although the amount of young pension recipients nevertheless remained relatively small, their increased number could have affected the comparisons. Furthermore, partial fixed-term disability pensions were excluded, as people receiving a partial disability pension usually remain in part-time work. Overall, the number of fixed-term pension recipients in this study was 10,177 in 2006 and 7918 in 2015.

### 2.2. Outcome Measures

Each fixed-term disability pension recipient was followed for four years from the beginning of the pension using the pension register and the shared employment register of the pension insurance companies. Transferring to a permanent disability pension and returning to work were used as the main outcomes of interest. Permanent disability pension included both full and partial pensions. Return to work was determined by the beginning of the first employment contract lasting for at least 4 weeks after the fixed-term disability pension had ended.

In addition, four other possible outcome states were defined. The first one included the unemployed and the second consisted of those who transferred to old-age pension or died (both relatively rare events). The third outcome included those whose fixed-term disability pension had ended but who were not in any of the states mentioned above. This group included recipients of paternal benefits, students, and others who cannot be identified in the registers. The last group included those who continued receiving a fixed-term disability pension.

### 2.3. Explanatory Variables

Gender, age, educational level, diagnosis for the fixed-term disability pension and participation in vocational rehabilitation were used as explanatory variables.

Age at the beginning of the fixed-term disability pension was classified into the age groups of 25–34, 35–44, 45–54, and 55–62 years. Educational level was classified into those with primary education or no qualifications, secondary education, and tertiary education.

The primary diagnosis of the fixed-term disability pension was classified into mental disorders (ICD-10 Chapter F), musculoskeletal diseases (Chapter M), and other diseases (including injury).

Participation in vocational rehabilitation during the fixed-term disability pension was measured by the receipt of the rehabilitation increment that is an additional sum paid to disability pension retirees when they participate in vocational rehabilitation [12]. Only vocational rehabilitation offered by pension insurers, consisting mainly of work try-outs, job coaching and occupational re-education, was covered. A requirement for the fixed-term disability pension is a plan of ongoing treatment or rehabilitation. It can therefore be assumed that those who did not participate in vocational rehabilitation received other medical care or medical rehabilitation.

### 2.4. Confounders

Three occupational characteristics were used as confounders. Occupational class was measured by the person’s main activity at the end of the year preceding the fixed-term pension. If the information was missing, the status at the end of the previous year was used. Occupational class was classified into manual workers’ lower non-manual employees (such as nurses or office clerks); upper non-manual employees (such as dentists or lawyers); entrepreneurs (including self-employed persons and owners of companies with salaried employees); and others, consisting mainly of persons who were unemployed, students, and those whose occupation was unknown.

Private sector and public sector employees were separated based on the information of their pension insurer. Public sector employees consisted of employees working for municipalities, joint municipal authorities, and central government.

Employment status before the fixed-term disability pension was based on the employment register. As a disability pension is usually preceded by a sickness allowance period of one year, information on employment one year before the fixed-term disability pension started was used.

### 2.5. Statistical Methods

The cumulative incidence of permanent disability pension, return to work, and the four other states during the follow-up time of four years is first illustrated among those who started their fixed-term disability pension in 2006 or 2015.

The change in the rate of transferring to permanent disability pension and returning to work over the study period was then examined using the Fine and Gray competing risks regression model [13]. This method extends the Cox proportional hazard model to account for competing events by modelling the effect of the covariates on the subdistribution hazard. The resultant hazard ratios denote the relative change in the rate of occurrence of an event in subjects who have not yet experienced the event of interest (but may have experienced a competing event). In our study, transferring to a permanent disability pension and returning to work were treated as competing events. The analyses were made for the whole study population and stratified by the explanatory variables. The results are presented as subdistribution hazard ratios (with 95 percent confidence intervals) for the change in the rate of transferring to permanent disability pension or returning to work. The year 2006 was used as the reference category to which those starting their fixed-term disability pension in 2015 were compared. Hazard ratios larger than one imply an increased probability of the outcome and those smaller than one a decreased probability of the outcome.

After unadjusted analyses, the results were adjusted for gender, age, level of education, diagnosis, and the three occupational confounders to determine whether changes in the composition of the fixed-term disability pensioners affected the findings. To examine whether the selection of applicants to have a fixed-term rather than a permanent disability pension affected the findings, the analyses were weighted to represent all new disability pensioners (fixed-term or permanent) with respect to gender, age, and diagnosis. These three characteristics very strongly determine whether the disability pension is granted for a fixed term or permanently [8], and controlling just for them accounts relatively well for the effect of selection. The analyses were conducted using Stata 16.

## 3. Results

**Figure 1:**
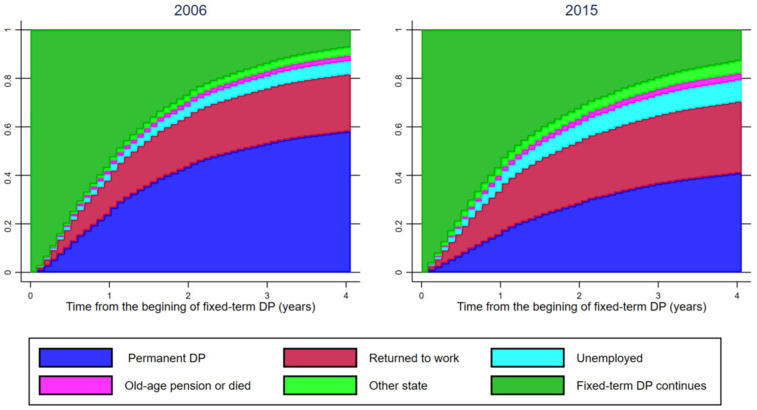
Cumulative incidence of permanent disability pension (DP), return to work, and the other labor market states over a four-year follow-up period among those who started receiving a fixed-term disability pension in 2006 and 2015.

Figure 1 shows the cumulative incidence of having a permanent disability pension, returning to work, and transferring to some other state during a four-year follow-up among those whose fixed-term disability pension started in 2006 or 2015. From 2006 to 2015, transferring to a permanent disability pension became clearly less common. Of those who started their fixed-term disability pension in 2006, 58 percent had received a permanent disability pension within four years. Ten years later, the corresponding figure was 41 percent. In contrast, the proportion of those who returned to work increased. After four years, 24 percent of those who started their fixed-term disability pension in 2006 had returned to work, while in 2015, the proportion was 30 percent. The return to work rate was fairly similar during the first two follow-up years (19 percent in 2006 and 21 percent in 2015) but, after that, the rate increased more among those who started their fixed-term disability pension in 2015. However, the returning to work increased less than transferring to a permanent disability pension decreased: an increasing proportion of the fixed-term disability pensioners became unemployed, moved to some other state, or continued on a fixed-term disability pension until the end of the follow-up period.

Table 1 shows the share of disability pensions granted for a fixed term and the distribution of the explanatory variables in 2006 and 2015. Overall, 49 percent of all disability pensions in 2006 were granted for a fixed term, while ten years later the share was 60 percent. The share of fixed-term disability pensions was larger for women, the younger age groups, those with a secondary or a tertiary education, and those whose pension was based on mental disorders or musculoskeletal diseases rather than other diseases. From 2006 to 2015, the share of fixed-term disability pensions increased in all subgroups. The increase was somewhat larger among the older people and those suffering from mental disorders.

The gender distribution of the fixed-term disability pensioners was similar in 2006 as in 2015, by chance equaling exactly 50 percent of men and women in both years (Table 1). The share of the youngest and the oldest fixed-term disability pensioners increased from 2006 to 2015, while the share of those with only a primary education decreased. The diagnoses were fairly equally distributed in the three categories, with no major changes between the study years. The proportion of those receiving vocational rehabilitation during their fixed-term disability pension was small but increased from 2006 to 2015.

Table 1 also shows the proportion of the fixed-term disability pensioners who transferred to a permanent disability pension and returned to work within four years by the explanatory variables. From 2006 to 2015, the probability of transferring to a permanent disability pension decreased almost equally for men and women, but the decrease was largest among the 45–54-year-olds and lowest among the 55–62-year-olds. In 2006, those with a lower education had a higher probability of transferring to a permanent disability pension, but by 2015, the differences between educational levels evened out. Transferring to a permanent disability pension decreased more among those with a fixed-term disability pension granted due to mental disorders or musculoskeletal diseases than among those suffering from other diseases. The decrease was also larger among those who had not participated in vocational rehabilitation. Return to work increased more among the 45–54-year-olds, those with a lower education, those with a fixed-term disability pension due to musculoskeletal diseases, and those participating in vocational rehabilitation. The effects of changes in the composition of fixed-term disability pensioners and the selection of applicants into fixed-term rather than permanent pensions on these outcomes are examined in more detail in later tables.

Occupational class, employment sector, and being employed before the onset of the fixed-term disability pension were used as confounders in further analyses. The proportion of manual workers decreased from 2006 to 2015, but, otherwise, the distributions of the occupational characteristics remained similar (Table 2). Transferring to a permanent disability pension was somewhat more common among the unemployed than among the other social classes, but the decrease over time was relatively similar in all groups. The differences were small in both years between private and public sector employees and those who were and were not employed before their fixed-term disability pension began. Return to work increased more among manual workers and lower white-collar employees than among the other occupational classes. The increase was similar among private and public sector employees but clearly larger among those who were employed before their fixed-term disability pension.

Table 3 shows the change in the relative rate of transferring to a permanent disability pension by the study variables, comparing those who started their fixed-term disability pension in 2015 with those who started in 2006. For the total study population, the rate of transferring to a permanent disability pension decreased by 40 percent. Adjusting for changes in the composition of fixed-term disability retirees very slightly decreased this estimate, whereas weighting the fixed-term disability retirees to represent all disability retirees in terms of age, gender, and diagnosis slightly increased it. The decrease in transferring to a permanent disability pension was larger in the younger age groups, those with lower education, and those suffering from mental disorders, but there were no differences by gender or receipt of vocational rehabilitation. Adjusting for changes in the composition had a larger effect on those with a higher education, those with a fixed-term disability pension due to musculoskeletal diseases, and those who had participated in vocational rehabilitation. Weighting had more of an effect on those with a primary education and those who had not participated in vocational rehabilitation. After adjusting for changes in the composition and controlling for selection, the decrease in the rate of transferring to a permanent disability pension was similar for all educational levels. In contrast, a difference emerged between those participating and not participating in vocational rehabilitation: after adjustment and weighing, the decrease was larger among those who had received vocational rehabilitation.

From 2006 to 2015, the return to work rate increased by 23 percent (Table 4). After adjusting for the composition of the fixed-term disability pensioners and controlling for the selection, the increase became even larger. The relative increase in the rate of return to work was larger in the older age groups, those with a lower education, those suffering from musculoskeletal diseases, and those who had participated in vocational rehabilitation. Adjusting for changes in the composition increased the return to work rate more among men, those aged 45–54, and those having a fixed-term disability pension due to musculoskeletal diseases. The adjustment also increased the change among those who had not received vocational rehabilitation but decreased it among those who had, thus narrowing the differences between these groups. The effect of weighting was similar in all studied groups.

## 4. Discussion

This study found that transferring from a fixed-term disability pension to a permanent disability pension considerably decreased in Finland during an era when the incidence of disability retirement decreased in general and the share of fixed-term pensions increased. Return to work increased, but not nearly as much as transferring to a permanent pension decreased. Fixed-term disability pensions also continued for longer than before, and an increasing part of the fixed-term disability pensioners ended up in some other state than permanent pension or work, most likely in unemployment.

Changes in the composition of fixed-term disability pensioners or in the selection of applicants into a fixed-term pension did not explain the differences between study years. On the contrary, the increase in the probability of return to work would have been even larger without such changes. In the older age groups and among those suffering from mental disorders, the proportion of fixed-term disability pensions has increased. Without such changes in these groups with a modest return to work rate, work resumption increased more than it actually did.

### 4.1. Differences by the Explanatory Variables

A slightly higher proportion of men than women transferred to a permanent disability pension, while return to work was slightly more common among women, but the differences in both outcomes were small. In previous studies, gender differences in the return to work have often been inconsistent [14], or the results have been poorer among women [15]. Concerning both outcomes, the development over time was also fairly similar among men and women. Relatively speaking, however, return to work increased slightly more among men. This gender difference was accentuated by controlling for changes in composition and selection.

The probability of transferring from a fixed-term disability pension to a permanent disability pension increases strongly by age. For people under the age of 35, it is rare to receive a permanent disability pension directly or—as the current study shows—even after a period of a fixed-term disability pension. This is partly, but not entirely, due to differences in medical diagnoses between age groups [8]. Our study shows that, over time, transferring to a permanent disability pension decreased in all age groups, but in the younger age groups, the proportion of those who returned to work remained fairly stable. This is partly because a larger proportion of those in the younger age groups continue to receive a fixed-term pension even after four years. Being in another state that could not be specified (including students) also partly explains the low return to work rates among the younger age groups. Nevertheless, it seems that the older age groups have been able to better compensate their decreasing transfer to permanent disability pension by increasing their return to work.

From 2006 to 2015, the share of fixed-term disability pensioners with only a primary education decreased, while that of the secondary or tertiary educated increased. This is mainly because, in more recent birth cohorts, particularly the older workers are better educated than before since a large section of employees with only a primary education have reached the old-age pension age [16]. The rising educational level of fixed-term disability pensioners may affect changes in the transferring to a permanent disability pension and returning to work as, for example, the fixed-term disability pensioners with only a primary education may be a more select group than before. Transferring to a permanent disability pension decreased more among those with a lower education, but adjusting for changes in the composition and controlling for selection equalized the differences. In addition, the return to work increased more among those with a lower education, but this was not clearly affected by changes in composition or selection.

The decrease in the probability of transferring to a permanent disability pension was larger among those whose fixed-term disability pension was granted due to mental disorders or musculoskeletal diseases than among those suffering from other diseases. In contrast, return to work from a fixed-term disability pension due to mental disorders increased significantly less than for other diagnoses. Poorer return to work outcomes among those suffering from mental disorders have been observed in several previous studies [17,18]. Due to increasing cognitive demands and intensification of working life [19,20], it may be that mental problems have become even more disabling than before.

While the decrease in transferring to a permanent pension can be considered a positive development, more effective measures to support return to work among those suffering from mental disorders are needed. The low return to work rate among those suffering from mental disorders is all the more important since, in this disease group, it is extremely common that a disability pension is initially granted for a fixed term. In 2015, the share reached 75 percent. Changes in the composition of fixed-term disability retirees or selection of the applicants into a fixed-term rather than a permanent pension did not explain the poor return to work outcome among those with mental disorders.

Participating in vocational rehabilitation while receiving a fixed-term disability pension was rare but increased from 2006 to 2015. Transferring to a permanent disability pension was clearly less common and returning to work more common among those who participated in vocational rehabilitation than those who did not. This should not be interpreted as a causal effect of vocational rehabilitation, as rehabilitation is offered to those who are expected to most benefit from it. In fact, while many studies have shown benefits from vocational rehabilitation [21,22], several studies have also shown only modest effects when selection has been taken into account [23,24,25].

Over time, the divergence between those participating and those not participating in vocational rehabilitation increased in both transferring to a permanent disability pension and returning to work. The increasing volume of vocational rehabilitation may indicate that the proportion of persons who are expected to benefit from vocational rehabilitation has increased. As the return to work rates were clearly higher among those who received vocational rehabilitation, this suggests that the selection of participants for vocational rehabilitation has been successful.

### 4.2. Methodological Considerations

The study population included all those who started their full fixed-term disability pension in 2006 or 2015. Partial disability pensioners were excluded as they usually continue working part-time besides their pension [26], which makes studying their return to work behavior inappropriate. However, the share of partial fixed-term disability pensioners has slightly increased over time, which again may have affected their selection into a full fixed-term disability pension.

Our measure of return to work was based on cumulative incidence of returning to work for at least four weeks. When considering the results, it should be therefore noted that all of those who returned to work are not necessarily working anymore at the end of the four-year follow-up period or any other date beyond the initial month of returning to work. Thus, the long-term return to work outcomes are likely to be somewhat poorer than shown here.

Changes in the general macroeconomic situation may affect return to work rates because employment prospects of those with work ability problems are likely to be better when the macroeconomic situation is favorable [27]. The overall employment and unemployment rates in Finland were relatively similar in 2006 and in 2015 [28], but the financial crisis in 2008 took place in the middle of the first follow-up period. Between 2006 and 2015, there were no changes in the legislation that could explain the results.

## 5. Conclusions

Transferring from a fixed-term disability pension to a permanent disability pension has decreased considerably, but the decrease is only partly reflected in an increased return to work. For an increasing proportion, the fixed-term disability pension continues for a longer time, and many fixed-term disability retirees end up in unemployment or some other state outside employment. Consistent with this, a recent systematic review found that although changes in the eligibility criteria for disability benefits often reduced the number of recipients of these benefits, there was no firm evidence of an increase in employment [29]. Among the younger and those suffering from mental disorders, return to work improved the least. Further efforts are needed to support work resumption, particularly in these groups.

Return to work increased more quickly during the first two follow-up years, but after that, it slowed down somewhat. The share of fixed-term disability pensions that continue for more than two years has increased, and in 2015, more than one-third exceeded that length. Extending the fixed-term disability pensions may be reasonable, as return to work has slightly increased. However, a drawback is that ending up in some other state has also increased. Therefore, decision makers must consider what is an acceptable balance between a slightly increasing return to work rate and dropping out from the disability pension but resting on unemployment and other benefits.

## Figures and Tables

**Table 1:** The share of fixed-term disability pensions (DP) of all disability pensions granted in 2006 and 2015, the distribution of fixed-term disability pensioners, and the proportion of those who transferred to permanent disability pension and returned to work (RTW) by main study variables.

	Share of Fixed-Term DP (%)		Distribution (%)		Permanent DP within 4 Years (%)		RTW within 4 Years (%)	
	2006	2015	2006	2015	2006	2015	2006	2015
Gender								
Men	45	55	50	50	60	44	22	28
Women	54	66	50	50	56	38	25	31
Age								
25–34	89	93	10	16	22	12	36	34
35–44	80	87	22	19	37	22	34	38
45–54	63	75	42	35	63	41	22	32
55–62	26	35	26	30	81	69	12	19
Educational level								
Primary	40	51	31	22	59	37	17	24
Secondary	55	62	50	57	51	36	23	27
Tertiary	55	63	18	21	49	38	27	27
Diagnosis								
Mental disorders	60	75	38	37	57	37	19	22
Musculoskeletal diseases	50	59	32	29	58	40	29	39
Other diseases	38	48	30	34	59	46	24	30
Vocational rehabilitation								
No	..	..	91	87	61	44	21	25
Yes	..	..	9	13	30	21	50	60
Total	49	60	100	100	58	41	24	30

**Table 2:** Distributions of the occupational confounding variables and the probability of transferring to permanent disability pension (DP) and returning to work (RTW) by these variables in 2006 and 2015.

	Distribution (%)		Permanent DP within 4 Years (%)		RTW within 4 Years (%)	
	2006	2015	2006	2015	2006	2015
Occupational class						
Manual workers	38	31	55	37	25	33
Lower non-manual employees	23	26	52	35	26	32
Upper non-manual employees	8	8	50	36	28	31
Entrepreneurs	9	9	53	40	23	25
Unemployed	4	8	61	42	11	10
Other or unknown	18	18	50	34	9	11
Employment sector						
Private	72	71	53	36	21	26
Public	28	29	53	39	23	27
Employment before FTDP						
No	36	39	52	37	12	13
Yes	64	61	53	37	27	34
Total	100	100	58	41	24	30

**Table 3:** Change in the rate of transferring to permanent disability pension by the study variables, comparing those starting their fixed-term disability pension in 2015 with those who started their fixed-term disability pension in 2006.

	Unadjusted ^1^	Adjusted ^2^	Adjusted and Weighted ^3^
Total	0.60 (0.58–0.63)	0.57 (0.55–0.60)	0.63 (0.60–0.67)
Gender			
Men	0.63 (0.59–0.67)	0.59 (0.55–0.63)	0.65 (0.60–0.70)
Women	0.58 (0.54–0.61)	0.55 (0.52–0.59)	0.62 (0.57–0.66)
Age			
25–34	0.51 (0.41–0.63)	0.49 (0.39–0.60)	0.49 (0.40–0.61)
35–44	0.53 (0.47–0.60)	0.52 (0.46–0.59)	0.53 (0.47–0.61)
45–54	0.51 (0.48–0.55)	0.50 (0.47–0.54)	0.51 (0.48–0.55)
55–62	0.68 (0.63–0.72)	0.68 (0.64–0.73)	0.70 (0.65–0.75)
Educational level			
Primary	0.53 (0.49–0.58)	0.54 (0.50–0.59)	0.63 (0.56–0.70)
Secondary	0.62 (0.59–0.66)	0.57 (0.53–0.60)	0.62 (0.58–0.68)
Tertiary	0.71 (0.64–0.78)	0.60 (0.54–0.67)	0.64 (0.56–0.73)
Diagnosis			
Mental disorders	0.53 (0.50–0.57)	0.55 (0.51–0.59)	0.60 (0.54–0.65)
Musculoskeletal diseases	0.59 (0.55–0.64)	0.51 (0.47–0.55)	0.56 (0.52–0.62)
Other diseases	0.69 (0.64–0.74)	0.65 (0.61–0.70)	0.69 (0.63–0.76)
Vocational rehabilitation			
No	0.62 (0.59–0.65)	0.59 (0.57–0.62)	0.66 (0.62–0.70)
Yes	0.64 (0.54–0.77)	0.50 (0.41–0.61)	0.49 (0.38–0.62)

^1^ The estimate is the subdistribution hazard ratio (95% confidence interval). ^2^ Adjusted for the other study variables and the confounders. ^3^ Weighted to correspond to the distributions of gender, age, and diagnosis of all disability pensions.

**Table 4:** Change in the rate of return to work by the study variables, comparing those starting their fixed-term disability pension in 2015 with those who started their fixed-term disability pension in 2006.

	Unadjusted ^1^	Adjusted ^2^	Adjusted and Weighted ^3^
Total	1.23 (1.15–1.30)	1.32 (1.25–1.40)	1.43 (1.33–1.54)
Gender			
Men	1.27 (1.16–1.38)	1.44 (1.33–1.57)	1.52 (1.36–1.69)
Women	1.19 (1.10–1.29)	1.23 (1.13–1.33)	1.34 (1.22–1.47)
Age			
25–34	0.88 (0.76–1.02)	0.98 (0.85–1.13)	0.97 (0.84–1.13)
35–44	1.06 (0.94–1.19)	1.11 (1.00–1.24)	1.12 (1.01–1.26)
45–54	1.37 (1.24–1.51)	1.51 (1.38–1.66)	1.53 (1.39–1.69)
55–62	1.71 (1.47–1.98)	1.72 (1.47–2.00)	1.71 (1.45–2.03)
Educational level			
Primary	1.53 (1.36–1.73)	1.62 (1.43–1.84)	1.72 (1.47–2.02)
Secondary	1.22 (1.13–1.32)	1.30 (1.20–1.40)	1.39 (1.27–1.52)
Tertiary	1.06 (0.94–1.19)	1.13 (1.00–1.28)	1.25 (1.08–1.45)
Diagnosis			
Mental disorders	1.10 (0.98–1.23)	1.12 (1.01–1.25)	1.17 (1.04–1.32)
Musculoskeletal diseases	1.34 (1.22–1.47)	1.51 (1.37–1.65)	1.58 (1.43–1.76)
Other diseases	1.24 (1.12–1.38)	1.30 (1.17–1.44)	1.44 (1.26–1.64)
Vocational rehabilitation			
No	1.13 (1.05–1.21)	1.25 (1.17–1.34)	1.33 (1.23–1.45)
Yes	1.46 (1.28–1.66)	1.35 (1.20–1.53)	1.46 (1.27–1.70)

^1^ The estimate is subdistribution hazard ratio (95% confidence interval).^2^ Adjusted for the other study variables and the confounders.^3^ Weighted to correspond to the distributions of gender, age, and diagnosis of all disability pensions.

## Data Availability

The data were compiled from administrative registers. Due to data protection regulations, the data cannot be shared publicly. As such, all researchers who wish to have access to the data are requested to contact the Finnish Centre for Pensions https://www.etk.fi/en/research-statistics-and-projections/research/research-cooperation/ (accessed on 26 April 2021) or the authors directly.
